# Supplementary material for: Autophagy receptor NDP52 alters DNA conformation to modulate RNA polymerase II transcription
Source: Nat Commun. 2023 May 18;14:2855. doi: 10.1038/s41467-023-38572-9 (PMC10195817; doi:10.1038/s41467-023-38572-9)
Supplement: Supplementary file 1 — Supplementary Information [file 41467_2023_38572_MOESM1_ESM.pdf]

SUPPLEMENTARY INFORMATION

Autophagy receptor NDP52 alters DNA conformation to modulate RNA Polymerase II transcription

Ália dos Santos, Daniel E. Rollins, Yukti Hari-Gupta, Hannah McArthur, Mingxue Du, Sabrina Yong Zi Ru, Kseniia Pidlisna, Ane Stranger, Faeeza Lorgat, Danielle Lambert, Ian Brown, Kevin Howland, Jesse Aaron, Lin Wang, Peter J. I. Ellis, Teng-Leong Chew, Marisa Martin-Fernandez, Alice L. B. Pyne, Christopher P. Toseland

Supplementary Figures

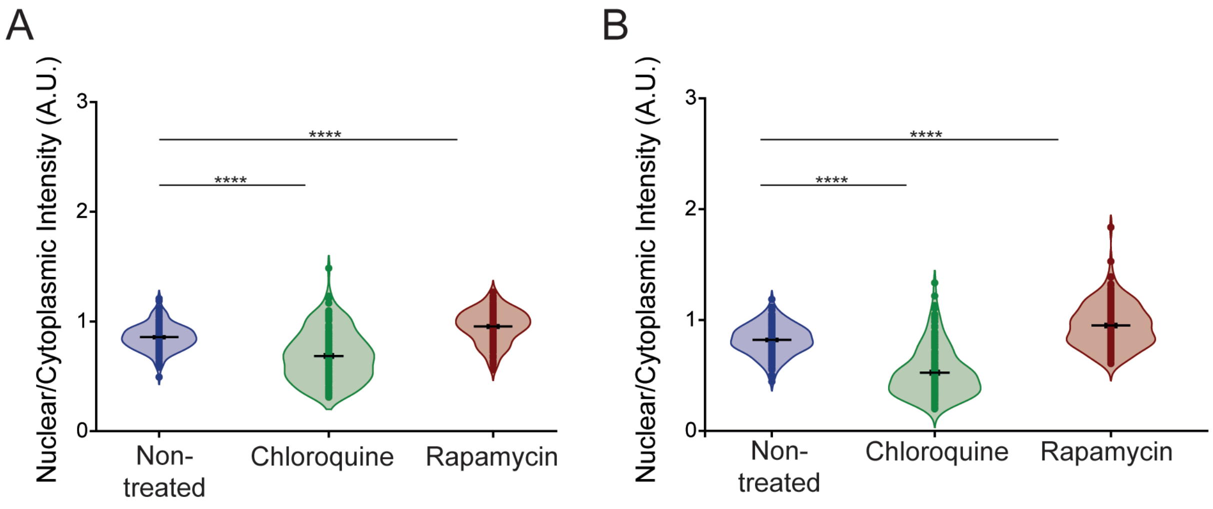

Supplementary Figure 1: Impact of autophagy on NDP52 nuclear localisation. Nuclear:cytoplasmic fluorescence intensity for NDP52, following inhibition or stimulation of autophagy following treatment with chloroquine and rapamycin, respectively. A and B represent two independent experiments with n=50 cells, Line represents mean. ****p<0.0001 by two-tailed t-test. (AU = Arbitrary units). Raw data is available in the Source Data file.

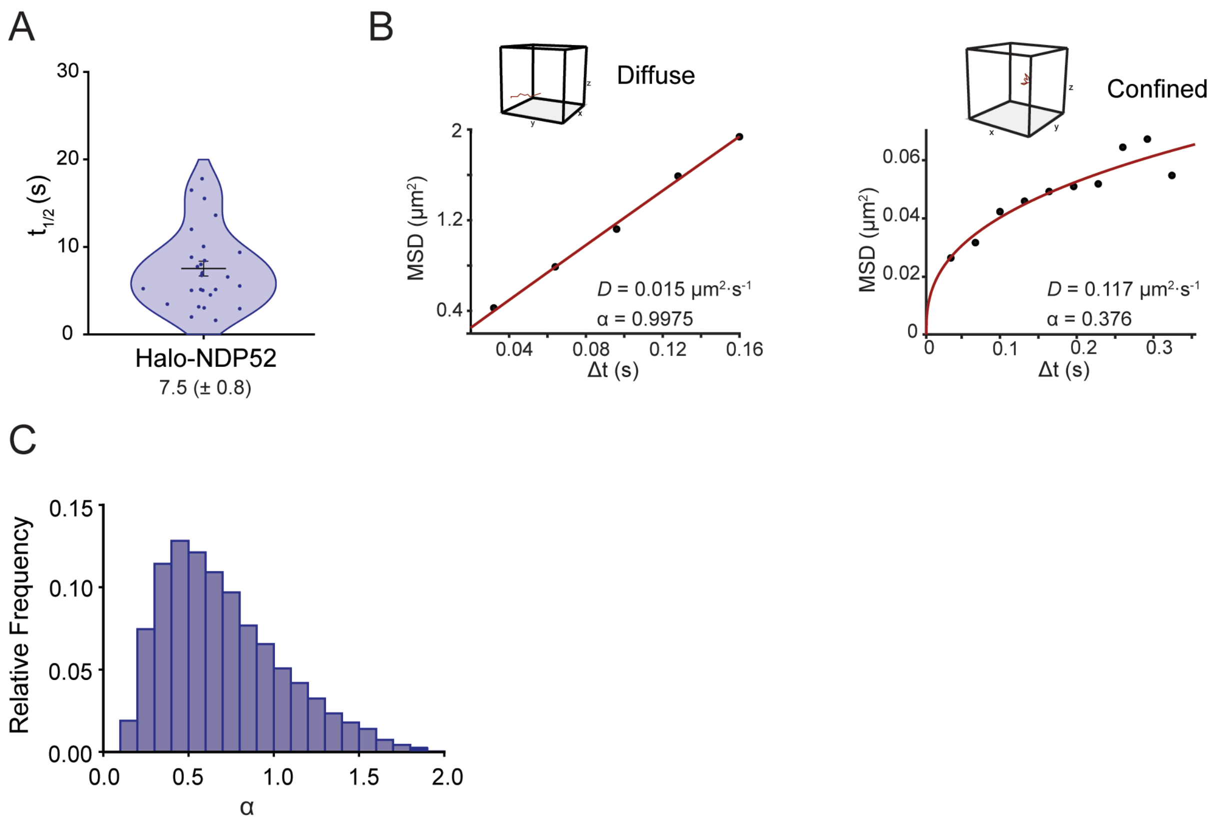

Supplementary Figure 2: Nuclear dynamics of NDP52. (A) Violin plot of recovery time ($t_{1/2}$) calculated from FRAP data for Halo NDP52. Each individual point represents one cell. Mean ± SEM value is shown. n = 27 cells. **(B)** Example MSD curves extracted and fitted from acMFM single-molecule tracks. 3D trajectory of each example is shown above the graph and calculated values for diffusion coefficient (D) and anomalous diffusion (α) are shown. **(C)** Histogram of calculated α values for all molecules using acMFM. Graph represents values for 14322 molecules from 51 cells. Raw data is available in the Source Data file.

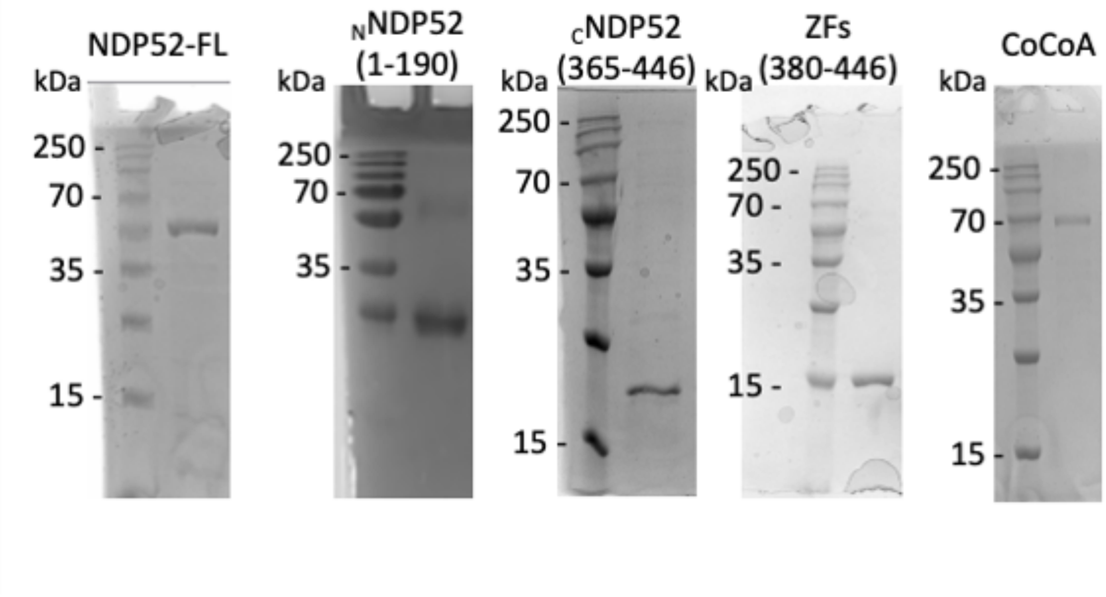

Supplementary Figure 3: SDS-PAGE analysis for proteins used in this study.

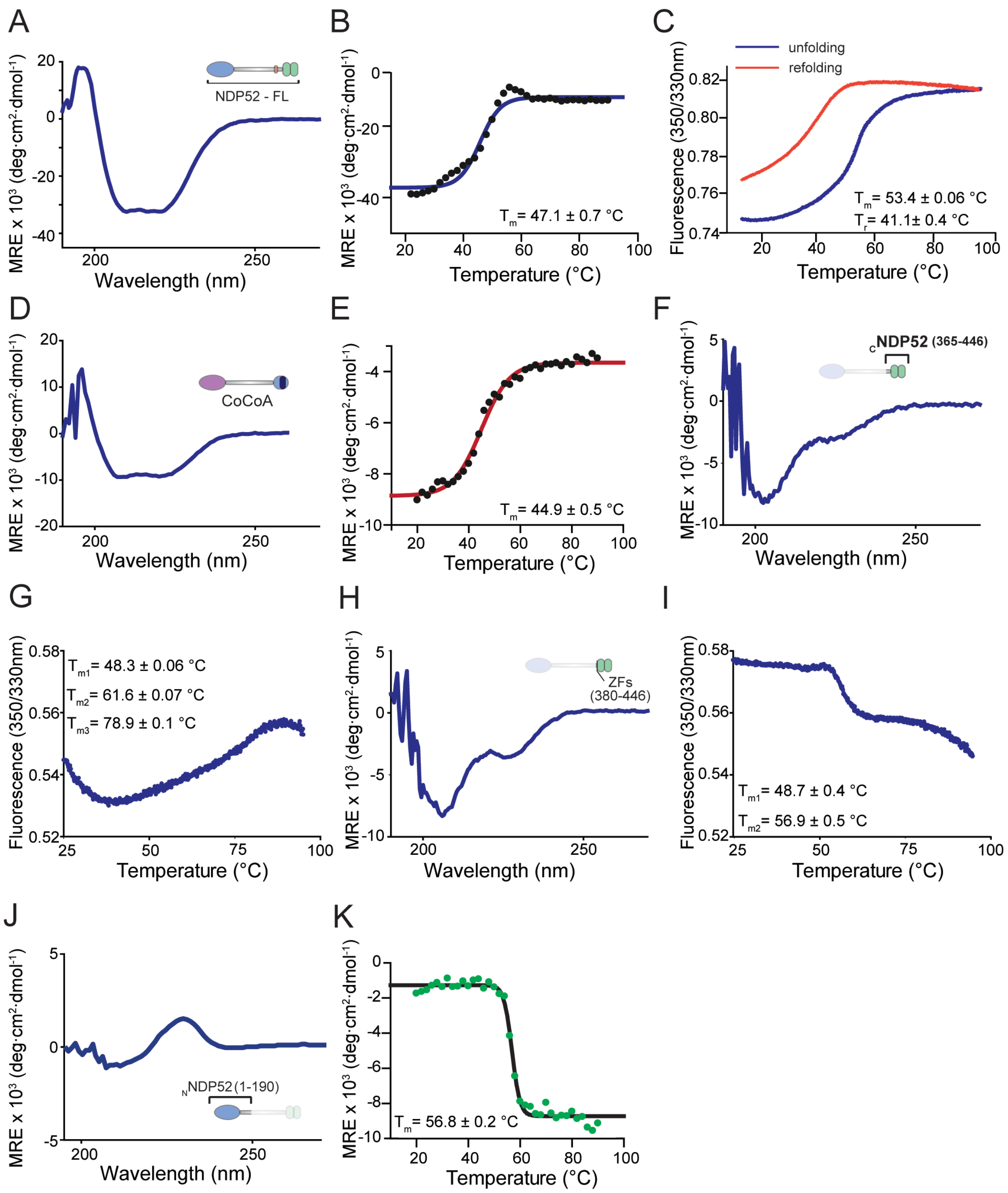

Supplementary Figure 4: Folding and stability of NDP52 and CoCoA recombinant constructs. (A) Circular Dichroism (CD) spectra of NDP52-FL. **(B)** Thermal denaturation of NDP52-FL following MRE values at 222 nm wavelength. **(C)** Nano-Differential Scanning Fluorimetry (nano-DSF) curves for NDP52 thermal denaturation and refolding. **(D)** CoCoA CD spectra. **(E)** Thermal denaturation of CoCoA, following MRE values at 222nm wavelength. **(F)** $_C$NDP52 CD spectra. **(G)** Nano-DSF for $_C$NDP52 during thermal denaturation. **(H)** ZFs CD spectra. **(I)** Nano-DSF for ZFs during thermal denaturation. **(J)** $_N$NDP52 CD spectra. **(K)** Thermal denaturation of $_N$NDP52, following MRE values at 215nm wavelength. Raw data is available in the Source Data file.

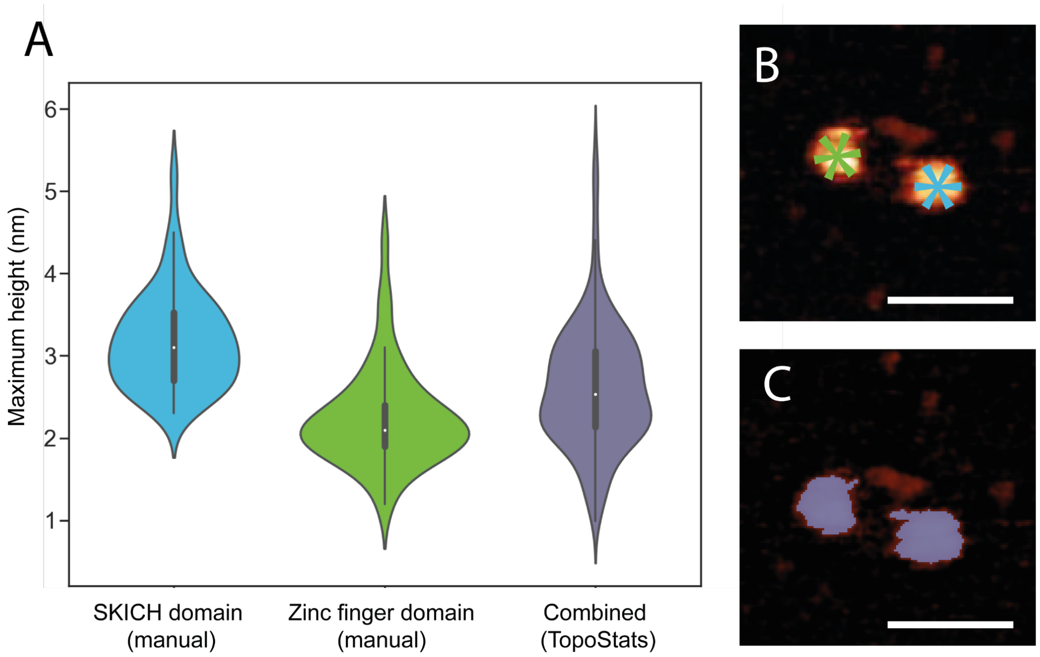

Supplementary Figure 5: Analysis and comparison of NDP52 domain heights measured by AFM (A) Maximum height measurements for the individual protein domains, as coloured in **(B)**. SKICH domain (blue) mean height: 3.2 nm. Zinc finger domain (green) mean height: 2.2 nm, along with automatic maximum height measurement based on mask detection of the domains (in purple) in **(C)** using TopoStats. The two populations have heights of 2.3 and 3 nm, respectively. (n = 202 molecules, Line represents Mean). Scale bars = 25 nm. Raw data is available in the Source Data file.

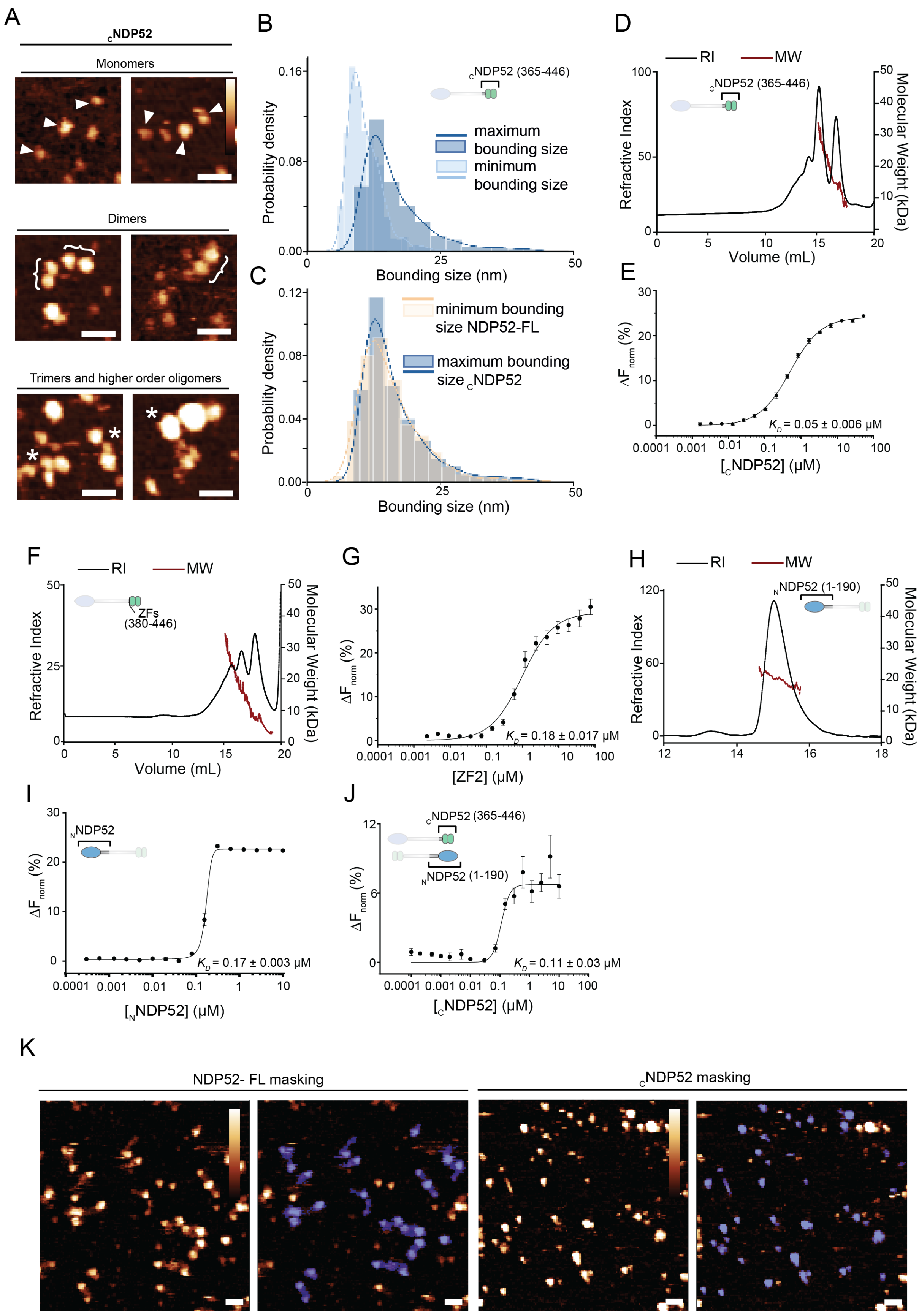

A
cNDP52
Monomers
Dimers
Trimers and higher order oligomers
B
cNDP52 (365-446)
maximum bounding size
minimum bounding size
Probability density
Bounding size (nm)
C
minimum bounding size NDP52-FL
maximum bounding size cNDP52
Probability density
Bounding size (nm)
D
RI
MW
cNDP52 (365-446)
Refractive Index
Molecular Weight (kDa)
Volume (mL)
E
ΔFnorm (%)
KD = 0.05 ± 0.006 μM
[cNDP52] (μM)
F
RI
MW
ZFs (380-446)
Refractive Index
Molecular Weight (kDa)
Volume (mL)
G
ΔFnorm (%)
KD = 0.18 ± 0.017 μM
[ZF2] (μM)
H
RI
MW
NNDP52 (1-190)
Refractive Index
Molecular Weight (kDa)
I
NNDP52
ΔFnorm (%)
KD = 0.17 ± 0.003 μM
[NNDP52] (μM)
J
cNDP52 (365-446)
NNDP52 (1-190)
ΔFnorm (%)
KD = 0.11 ± 0.03 μM
[cNDP52] (μM)
K
NDP52- FL masking
cNDP52 masking

Supplementary Figure 6: Oligomerisation of individual NDP52 domains. (A) Atomic force microscopy imaging (AFM) of $_{C}$NDP52 showing monomeric, dimeric and higher oligomeric forms of the protein on PLL. Scale bar = 25 nm. Height scale = 4.5 nm. **(B)** Histogram and kernel density estimate (KDE) plots of minimum and maximum bounding sizes, *i.e.*, length and width for $_{C}$NDP52 particles, calculated from masked molecules in AFM images (examples shown in K). Peaks in KDE plots were used to determine particle size (KDE max ± SD); minimum = 9 ± 3 nm, maximum = 13 ± 6. N = 1 909 molecules. **(C)** KDE distribution showing maximum bounding sizes of $_{C}$NDP52 (data shown in B) compared to minimum bounding size for NDP52-FL (data shown in Fig.4L and Supplementary Fig.6K). **(D)** SEC-MALS profile for the C-terminal of NDP52 ($_{C}$NDP52), which encompasses both zinc finger domains. Refractive index (RI) trace is shown in black, as well as the calculated molecular weight values, across the peaks (in red). **(E)** Microscale thermophoresis, showing oligomerisation of $_{C}$NDP52, with the calculated K_D displayed in the graph. Values plotted represent average ± SEM of three individual experiments. **(F)** SEC-MALS trace for ZFs. RI trace is shown in black and calculated molecular weight values are shown in red. **(G)** Microscale thermophoresis, showing oligomerisation of ZFs, with the calculated K_D displayed in the graph. Values represent average ± SEM of three individual experiments. **(H)** SEC-MALS trace for the N-terminal of NDP52 ($_{N}$NDP52), comprising the SKICH domain and a small portion of the coiled-coil region. RI trace is shown in black and calculated molecular weight values are shown in red. **(I)** Microscale thermophoresis, showing oligomerisation of $_{N}$NDP52, with the calculated K_D displayed in the graph. Values plotted represent average ± SEM of three individual experiments. **(J)** MST curve showing interaction between labelled $_{N}$NDP52 titrated with $_{C}$NDP52, with the calculated K_D displayed in the graph. Values represent average ± SEM of three individual experiments. **(K)** AFM images of NDP52-FL and $_{C}$NDP52 on PLL with corresponding images showing molecules masked (in blue) and measured by Topostats. Scale bar = 25 nm. Height scale = 4.5 nm (scale bar inset). Raw data is available in the Source Data file.

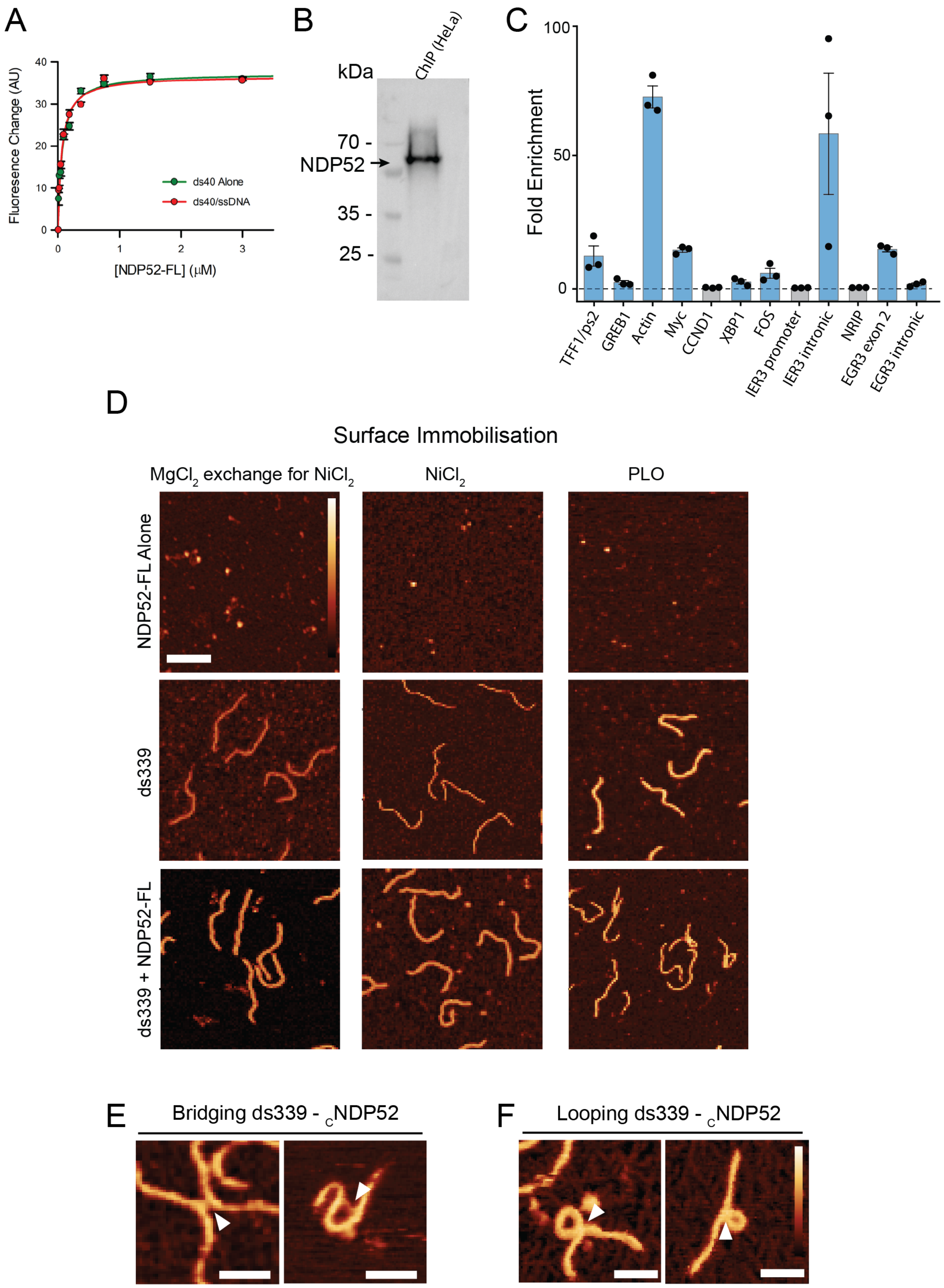
A
Fluoresence Change (AU)
[NDP52-FL] (μM)
ds40 Alone
ds40/ssDNA
B
ChIP (HeLa)
kDa
70 -
NDP52
35 -
25 -
C
Fold Enrichment
TFF1/ps2
GREB1
Actin
Myc
CCND1
XBP1
FOS
IER3 promoter
IER3 intronic
NRIP
EGR3 exon 2
EGR3 intronic
D
Surface Immobilisation
MgCl2 exchange for NiCl2
NiCl2
PLO
NDP52-FL Alone
ds339
ds339 + NDP52-FL
E
Bridging ds339 - CNDP52
F
Looping ds339 - CNDP52

Supplementary Figure 7: NDP52 binding to DNA in cells and *in vitro*. **(A)** Fluorescence spectroscopy titrations of NDP52-FL against 40bp fluorescein amidite double-stranded DNA (ds40) in the presence and absence of single stranded DNA (40 bases). K_D <100 nM. Values plotted represent average ± SEM of three individual experiments. (AU = Arbitrary units). **(B)** Western blot showing presence of NDP52 in chromatin-enriched ChIP sample. **(C)** NDP52 ChIP-qPCR against *loci* specified in the label. Enriched *loci* are shown in blue. Values represent mean ± SEM of three individual experiments. **(D)** AFM images of NDP52-FL and DNA (ds339) co-adsorbed individually and together on $MgCl_2$, $NiCl_2$ and poly-L-ornithine (PLO). Scale bar 50 nm. Height scale = 4.5 nm. **(E)** AFM images of ds339 co-adsorbed with $_C$NDP52. Arrowheads show $_C$NDP52 bridging DNA strand(s). Scale bars = 25 nm. **(F)** AFM images of ds339 co-adsorbed with $_C$NDP52. Arrowheads show DNA looping induced by $_C$NDP52. Scale bars = 25 nm and Height scale = 4.5 nm. Raw data is available in the Source Data file.

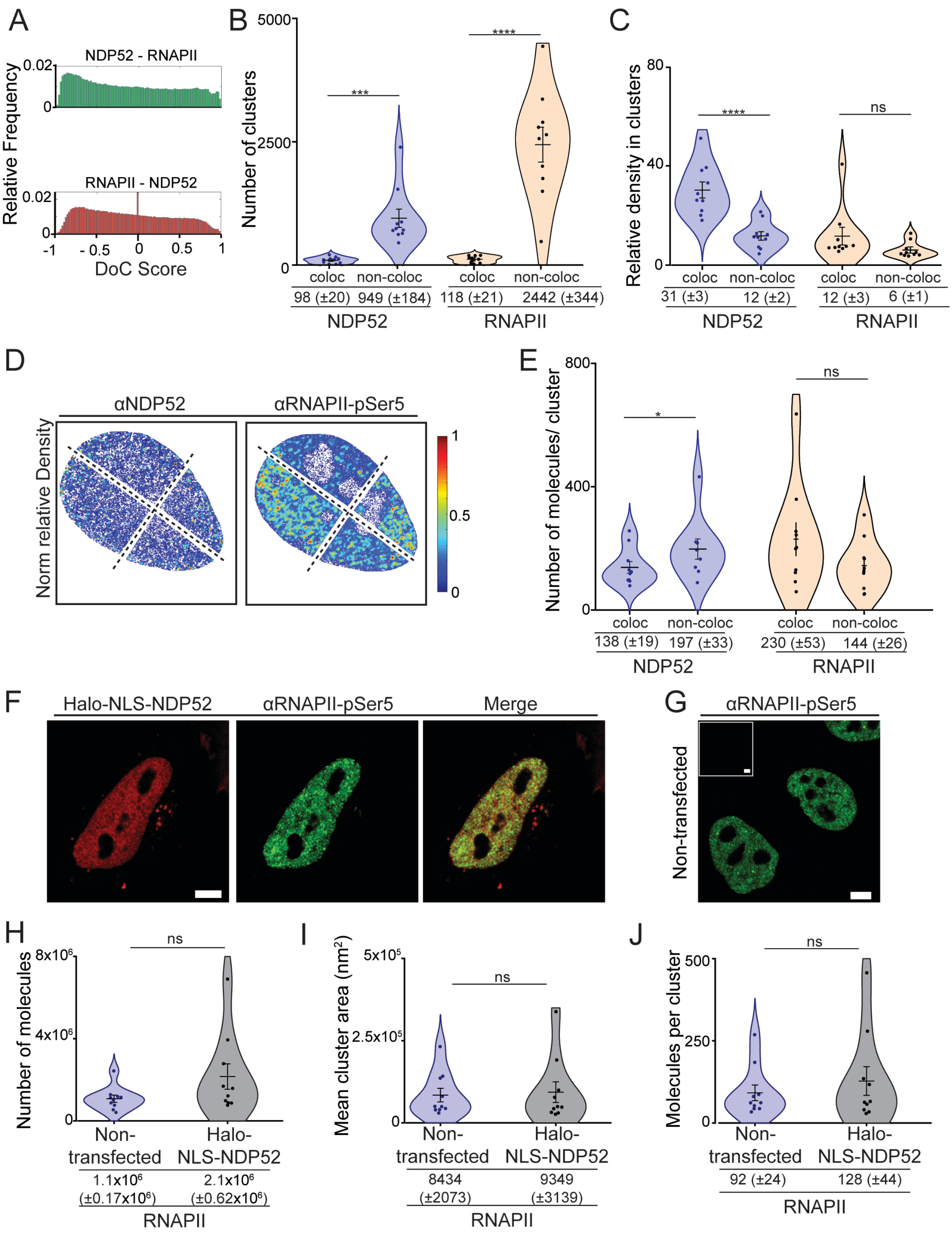

Supplementary Figure 8: Colocalisation of NDP52 with RNAPII-pSer5. (A) DoC score histogram for example STORM rendering in Figure 7C and colocalisation map shown in Figure 8D. DoC score of 1 represents perfect colocalisation between molecules, and DoC score -1 represents segregation. DoC score of 0.4 was used as colocalisation threshold. **(B)** Number of clusters considered colocalised or non-colocalised for both NDP52 and RNAPII-pSer5. (n = 10 cells) **(C)** Relative density of molecules in colocalised and non-colocalised NDP52 or RNAPII-pSer5 clusters. (n = 10 cells) **(D)** Example of normalised relative density heat maps for NDP52 and RNAPII-pSer5. Heat maps correspond to the same cell shown in Figure 8B

and D. Red represents regions of high molecular density and blue of low molecular density. **(E)** Mean number of molecules per colocalised or non-colocalised clusters for NDP52 or RNAPII-pSer5. n = 10 cells **(F)** Confocal images of HeLa cell transiently expressing Halo-NLS-NDP52 and immunofluorescently stained with RNAPII-pSer5. Scale bar = 5µm. **(G)** Confocal image of non-transfected HeLa cell with immunofluorescent staining of RNAPII-pSer5. Scale bar = 5µm. **(H)** Number of RNAPII-pSer5 molecules in non-transfected or transiently expressing Halo-NLS-NDP52 HeLa cells. **(I)** Mean cluster area in nm^2 for RNAPII-pSer5 molecules in non-transfected or transiently expressing Halo-NLS-NDP52 HeLa cells. **(J)** Number of molecules per RNAPII-pSer5 cluster in in non-transfected or transiently expressing Halo-NLS-NDP52 HeLa cells. Mean ± SEM values are shown. Each point represents the average value per cell. n = 10 cells (Non-transfected) n = 10 cells (Halo-NLS-NDP52). *p<0.05; ***p<0.001; ****p<0.0001 by two-tailed t-test. Raw data is available in the Source Data file.

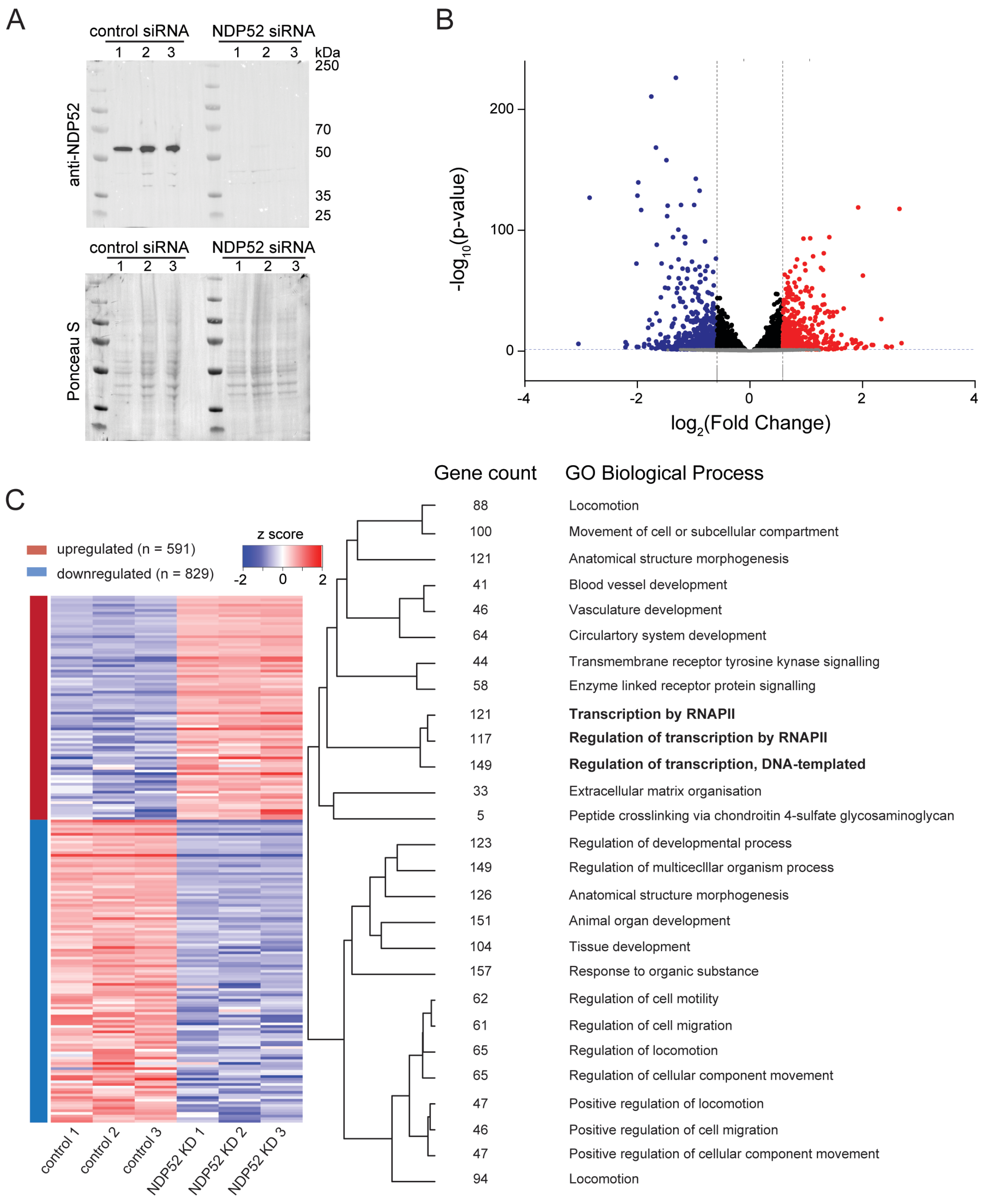

Supplementary Figure 9: Impact of NDP52 knockdown in gene expression in HeLa cells. (A) Western blot showing NDP52 KD replicates for RNA-Seq. On top, immunoblot against NDP52 is shown and, on the bottom panel, the same blot stained with Ponceau S is shown as loading control. **(B)** Volcano plot of differentially expressed genes following NDP52 KD. P values calculated by Wald test. **(C)** Heat map showing top down and upregulated genes in HeLa cells showing z-score values for control siRNA and NDP52 KD, obtained from three independent experiments. Dendrogram and enriched GO terms are shown. Number of genes differentially expressed for each GO term is also displayed. All GO terms shown have FDR<0.01. For details see Supplementary Table 5.

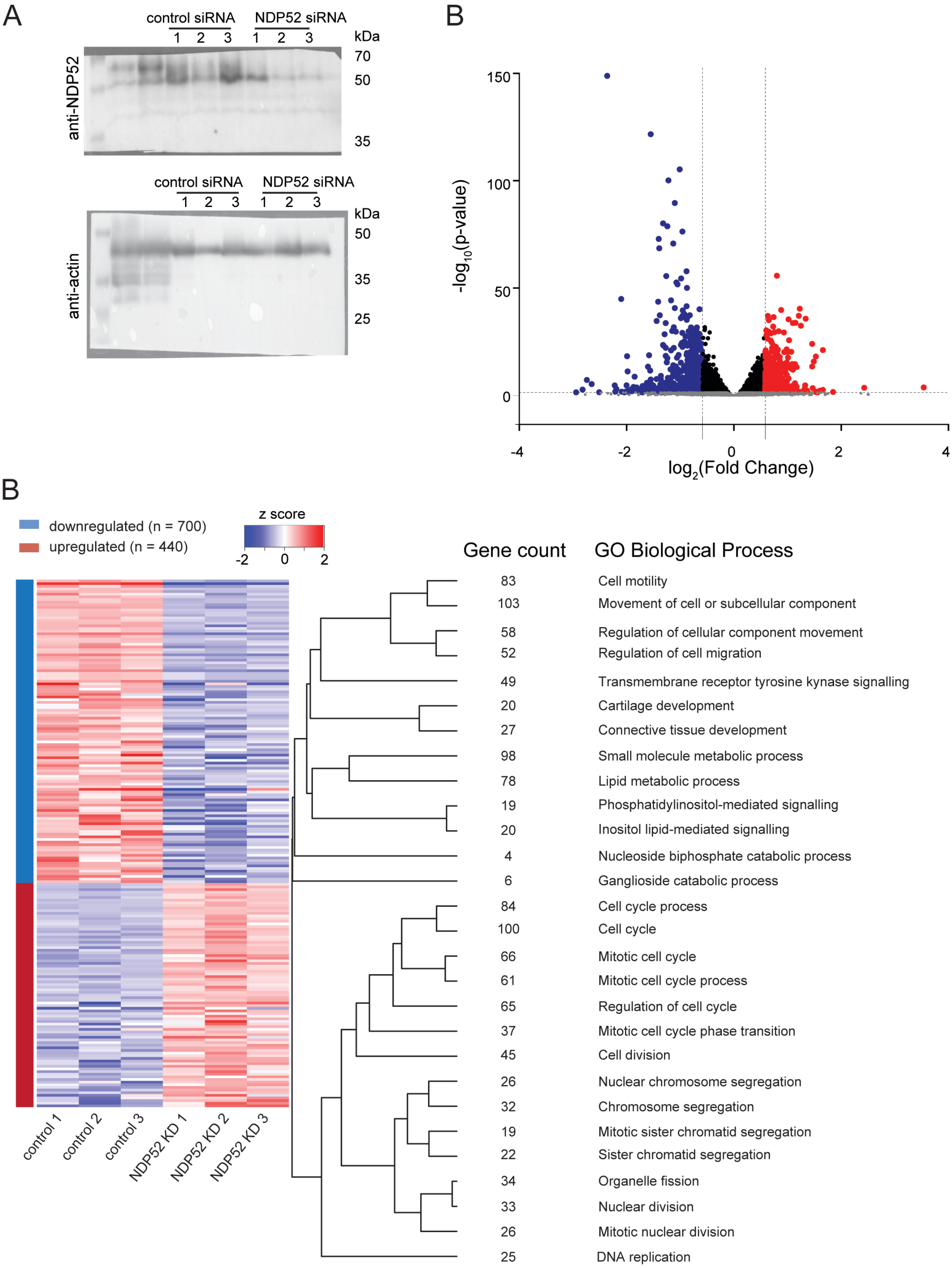

Supplementary Figure 10: Impact of NDP52 knockdown in gene expression in MCF-7 cells. (A) Western blot showing NDP52 KD replicates for RNA-Seq. On top, immunoblot against NDP52 is shown and, on the bottom panel, the anti-actin immunoblot is shown. **(B)** Volcano plot of differentially expressed genes following NDP52 KD in MCF-7 cells. p values calculated by Wald test. **(C)** Heat map showing top down and upregulated genes and z-score

values for control siRNA and NDP52 KD from three independent experiments. Dendrogram and enriched GO terms are shown. Number of genes differentially expressed for each GO term is also displayed. All GO terms shown have FDR<0.01. For details see Supplementary Table 6.

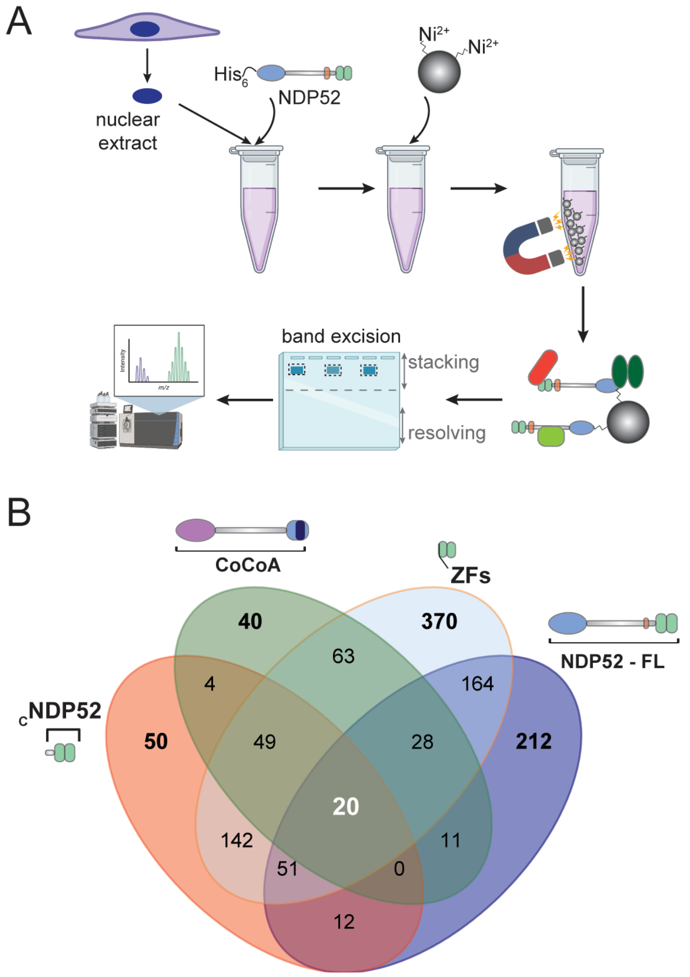

C

Hit	ZFs log2(FC)	ZFs p	known function
GATAD2B	∞	**	transcription
SWT1	∞	***	transcription
PARG	∞	**	DNA repair
WIZ	∞	**	transcription
SCMH1	∞	***	transcription
FANCA	∞	**	DNA repair
ARNTL	∞	***	transcription
RECQL5	∞	*	helicase
TTF2	∞	****	transcription
RERE	7.1	****	transcription
ARID1B	∞	***	chromatin regulation
KDM5C	∞	**	chromatin regulation
FUBP1	∞	*	transcription
SETDB1	∞	**	chromatin regulation
NELFE	∞	*	transcription
MED12	∞	***	transcription
TCF4	--	--	transcription
SKIV2L	∞	*	helicase

Supplementary Figure 11: Recombinant and endogenous NDP52 interactomes in non-treated and α-amanitin treated HeLa (A) Diagram depicting recombinant protein proteomics protocol. Briefly, following nuclear isolation from HeLa cells, recombinant constructs were incubated with nuclear extract. His-tagged constructs bound to interacting partners were pulled-down using magnetic Ni-NTA beads. Samples were run in SDS-PAGE gels and all proteins from each replicate were excised as a single band from the stacking portion of the polyacrylamide gel, for LC-MS/MS experiments. Created with BioRender.com **(B)** Venn diagram of hits found in label-free quantitative LC-MS/MS data for pull-downs of recombinant protein from HeLa nuclear extract. **(C)** Examples of top hits for NDP52 and CoCoA shown in Figures 8B and 8D, and their identification in ZFs proteomics data. log_2FC is relative to beads control. *FDR<0.05, **FDR<0.01, ***FDR<0.001, ****FDR<0.0001 by ANOVA.

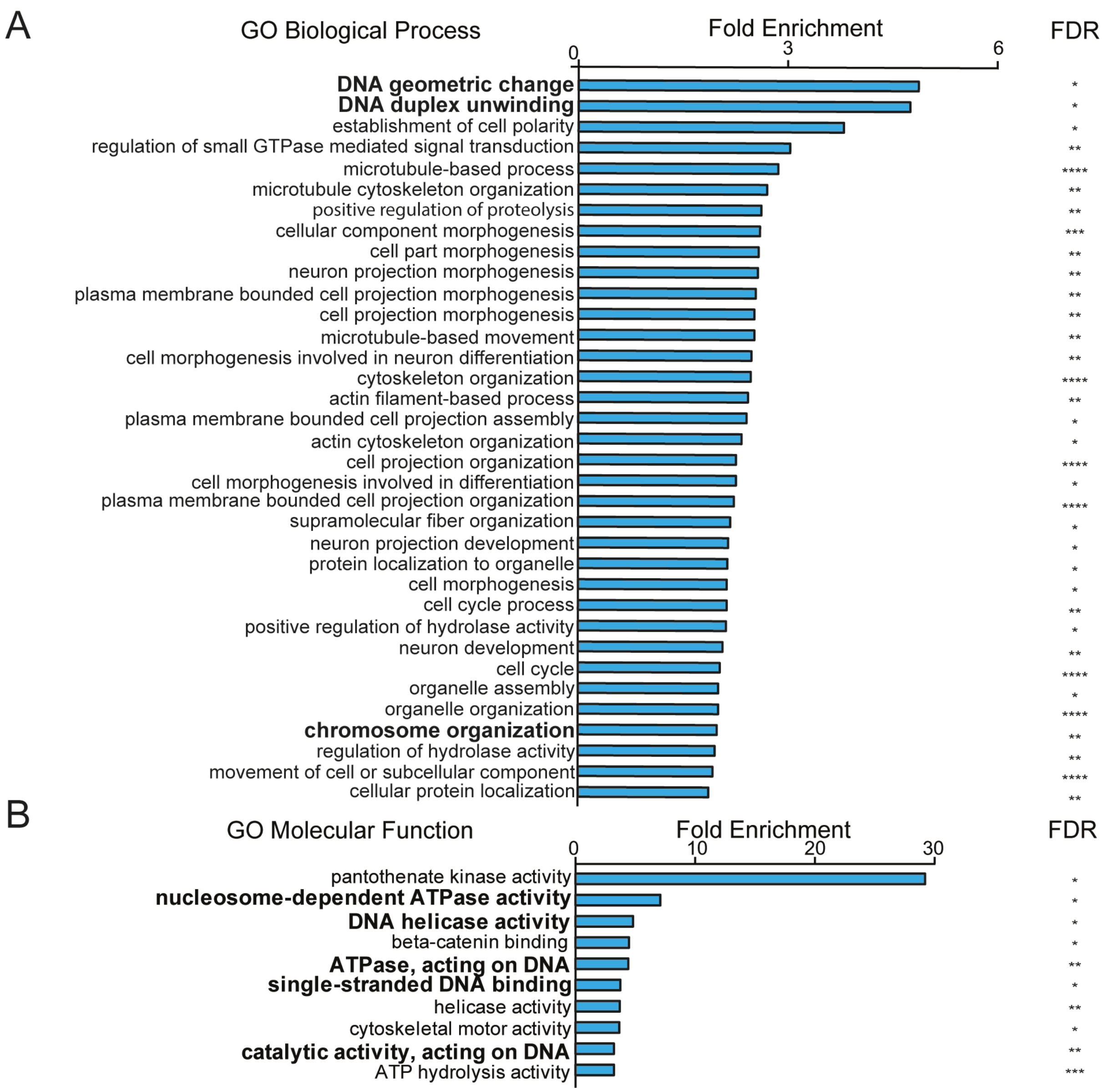

Supplementary Figure 12: GO Biological Process and Molecular Function analysis for NDP52-FL-interacting proteins in HeLa nuclear extract. (A) Top 35 Gene Ontology Biological Process annotation terms for NDP52-FL interactions. **(B)** Top 10 Gene Ontology molecular function terms for identified interactors of NDP52-FL. Fold enrichment and FDR values are shown. * FDR<0.05, ** FDR<0.01, *** FDR<0.001, **** FDR<0.0001. For full lists refer to Supplementary tables 7 and 8.

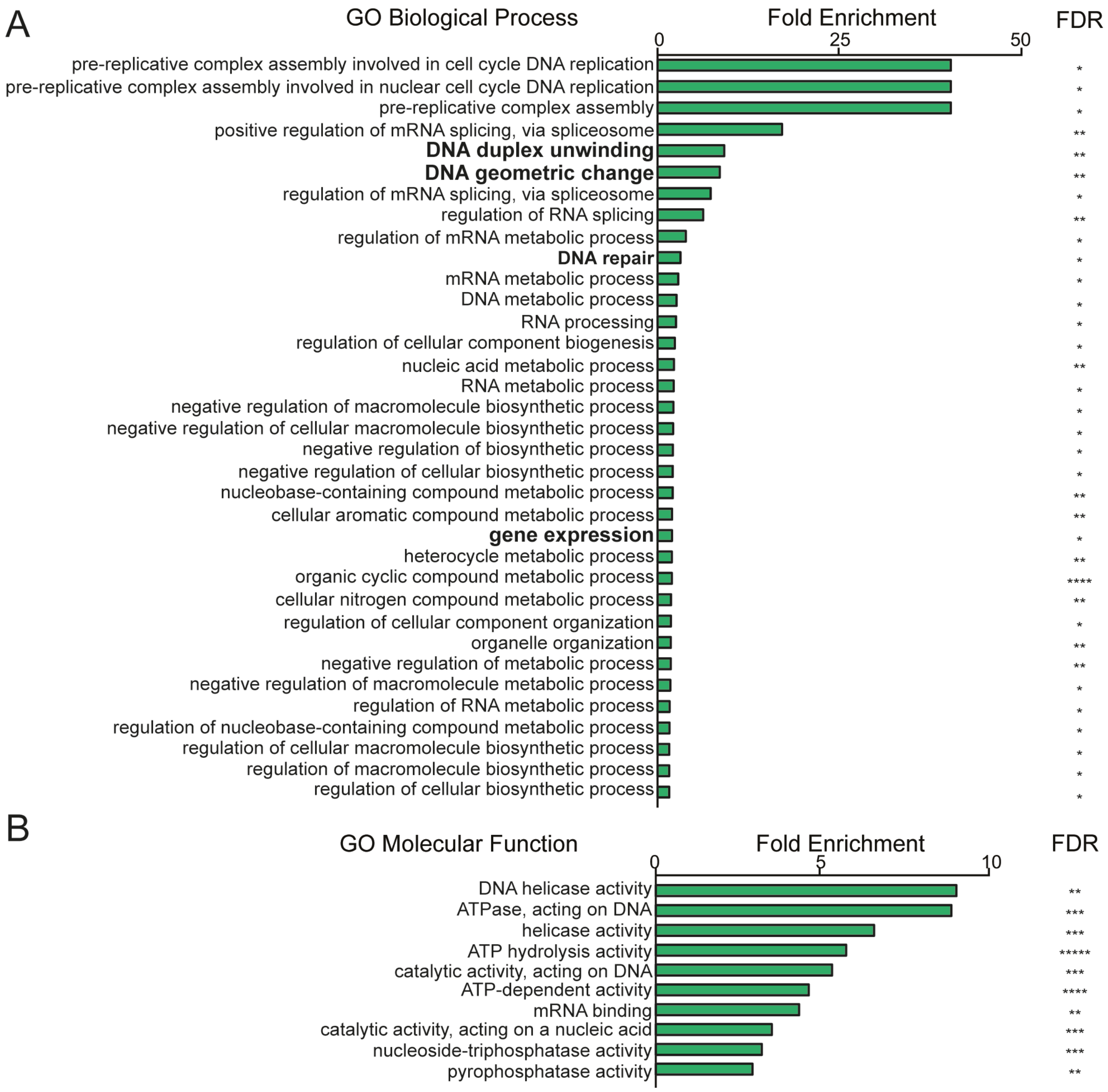

Supplementary Figure 13: GO Biological Process and Molecular Function analysis for CoCoA-interacting proteins in HeLa nuclear extract. (A) Top 35 Gene Ontology Biological Process annotation terms for CoCoA interactions. **(B)** Top 10 Gene Ontology molecular function terms for identified interactors of CoCoA. Fold enrichment and FDR values are shown. * FDR<0.05, ** FDR<0.01, *** FDR<0.001, **** FDR<0.0001. For full lists refer to Supplementary tables 9 and 10.

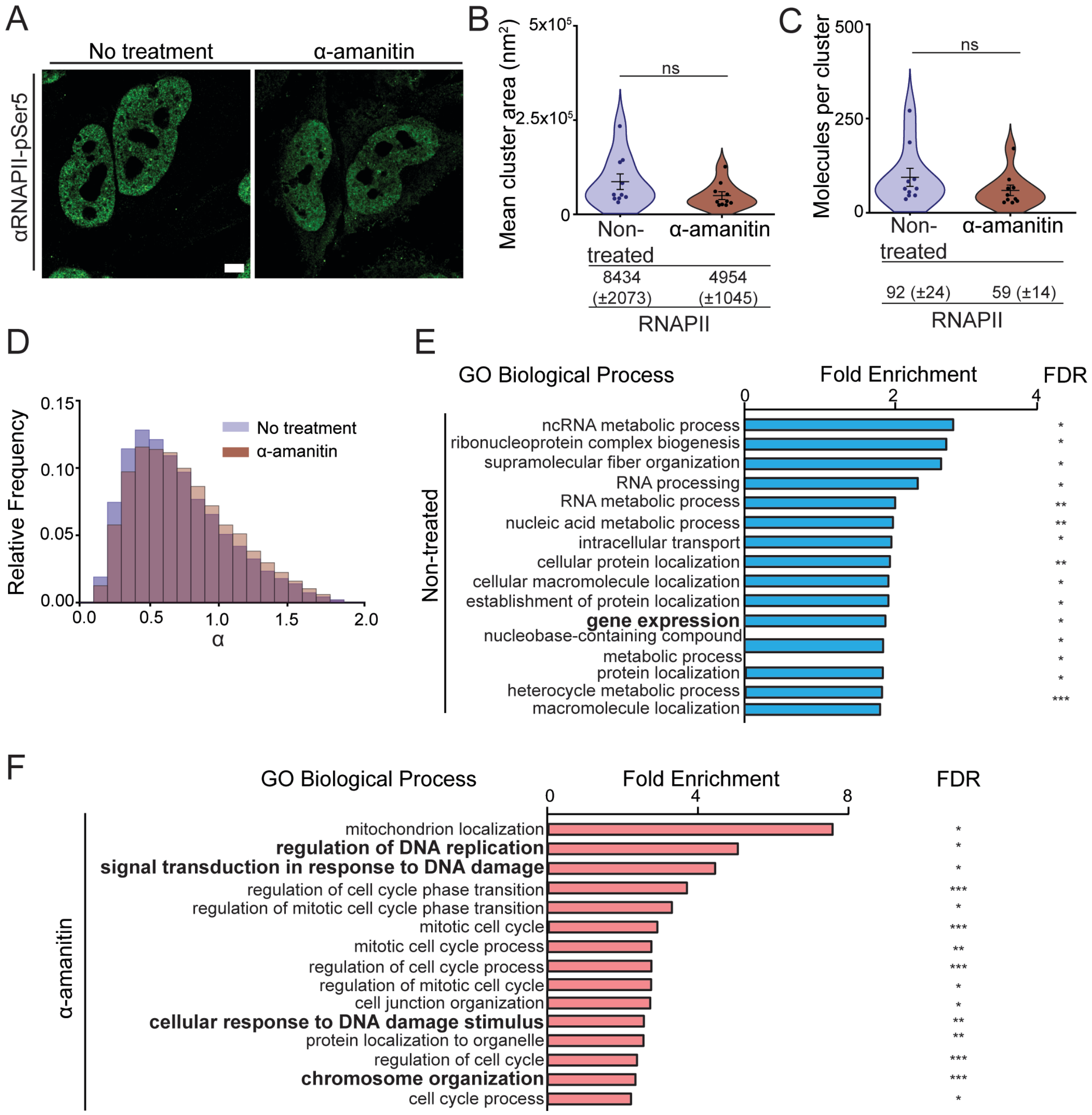

Supplementary Figure 14: Effect on transcription inhibition on the nuclear spatial organisation and dynamics of NDP52. (A) Confocal images of RNAPII-pSer5 in non-treated and α-amanitin treated HeLa cells. **(B)** Mean cluster area in nm^2 for RNAPII-pSer5 in non-treated and α-amanitin treated cells. ns= p>0.05 by two-tailed t-test. **(C)** Number of RNAPII-pSer5 per cluster in non-treated and α-amanitin treated cells. Mean ± SEM values are shown. Each point represents the average value per cell. *n* = 10 cells (non-treated) *n* = 10 cells (α-amanitin). ns= p>0.05 by two-tailed t-test. **(D)** Anomalous diffusion (α) histograms for cells transiently expressing Halo-NLS-NDP52, non-treated or following α-amanitin treatment. Histogram represents values for 14322 molecules from 51 cells non-treated condition (in blue – same as shown in Supplementary Figure 2C) and 14 492 molecules from 50 cells for α-amanitin treatment (in red). **(E-F)** Changes in the interactome of endogenous NDP52 in non-treated and α-amanitin treated HeLa cells. **(E)** Top 15 Gene Ontology Biological Process annotation terms for NDP52 interactions in non-treated HeLa cells. **(F)** Top 15 Gene Ontology molecular function terms for identified for interactions of NDP52 following α-amanitin treatment. Fold enrichment and FDR values are shown. * FDR<0.05, ** FDR<0.01, *** FDR<0.001, **** FDR<0.0001. For full lists refer to Supplementary tables 11 and 12.

Supplementary Tables

Supplementary Table 1 – List of constructs.

**Construct**	**Source**
Human pET151 Halo-NDP52	Synthetic Gene
Human pET151 Halo-NLS-NDP52	Synthetic Gene
Human pET151 NDP52-FL (1-end)	Synthetic Gene
Human pET151 CoCoA (1-end)	Synthetic Gene
Human pET151 $_{C}$NDP52 (365-end)	Synthetic Gene
Human pET151 ZFs (380-end)	Synthetic Gene
Human pET151 $_{N}$NDP52 (1-190)	Synthetic Gene
Human pcDNA3.1 Halo-NLS-NDP52	Synthetic Gene

Supplementary Table 2 – ChIP-qPCR primers

Description	Oligonucleotide sequence
TFF1/PS2 Forward	CCATGTTGGCCAGGCTAGTC
TFF1/PS2 Reverse	ACAACAGTGGCTCACGGGGT
GREB1 Forward	GCAGTGAAAAAAAGTGTGGCAACTGGG
GREB1 Reverse	GACCCACAGAAATGAAAAGGCAGCAAACT
Actin Forward	GACCTTCAACACCCCAGCC
Actin Reverse	GTCACGCACGATTTCCCGCT
Myc Forward	GGCTCACCCTTGCTGATGCT
Myc Reverse	GCTCTGGGCACACACATTGG
CCND1 Forward	GCCAGACAACATTAGCCACA
CCND1 Reverse	CCCATTTCTTAGCTGGGTCA
XBP1 Forward	ATACTTGGCAGCCTGTGACC
XBP1 Reverse	GGTCCACAAAGCAGGAAAAA
FOS Forward	GGGCACTTACACACACATGC
FOS Reverse	ATGATACTCGCGAGCAGAGG
IER3 Promoter Forward	CCCCTGCTTCTTCTCAGTTG
IER3 Promoter Reverse	AAAAGATGCACGGATTGGAG
IER3 Intronic Forward	CGCCGAAGTCTCACACAGTA
IER3 Intronic Reverse	AGGAGAAGAAATGGGGAGGA
NRIP Forward	GACCTGGCCCAGATAGATCA
NRIP Reverse	TATAATGTAGGGGCGCAACC
EGR3 exon 2 Forward	GGTGCCTGAGAAGAGGTGAG
EGR3 exon 2 Reverse	CCATGTGGATGAATGAGGTG
EGR3 Intronic Forward	TCACGTACCACACACACACG
EGR3 Intronic Reverse	TCCGGGTCTGAAACTACCTG
GADPH qPCR Forward	GTCTCCTCTGACTTCAACAGCG
GADPH qPCR Reverse	ACCACCCTGTTGCTGTAGCCAA
HBB qPCR Forward	CACCTTTGCCACACTGAGTGAG
HBB qPCR Reverse	CCACTTTCTGATAGGCAGCCTG

Supplementary Table 3 – List of DNA constructs.

Description	Oligonucleotide sequence
ds15A 3'FAM	TTAGTTGTTCTCTGG*
ds15B	CCAGAGAACAACTAA
ds40A 3'FAM	TTAGTTGTTCGTAGTGCTCGTCTGGCTCTGGATTACCCGC*
ds40B	GCGGGTAATCCAGAGCCAGACGAGCACTACGAACAACTAA
ss40 3'FAM	TTAGTTGTTCGTAGTGCTCGTCTGGCTCTGGATTACCCGC*
ds339	TATGTTGTGTTTTACAGTATTATGTAGTCTGTTTTTTATGCAAAATCTAATTTAATATATTGATATTTATATCATTTTACGTTTCTCGTTCAGCTTTTTTATACTAACTTGAGCGAAACGGGAAGGGTTTTCACCGATATCACCGAAACGCGCGAGGCAGCTGTATGGCGAAATGAAAGAACAAACTTTCTTGTACGCGGTGGTGAGAGAGAGAGAGAGATACGACTACTATCAGCCGGAAGCCTATGTACCGAGTTCCGACACTTTCATTGAGAAAGATGCCTCAGCTCTGTTACAGGTCACTAATACCATCTAAGTAGTTGATTCATAGTGACTGCA

Supplementary Table 4 – Adsorption methods for AFM imaging of DNA molecules in the presence and absence of NDP52-FL and $_{C}$NDP52.

Sample	Adsorption Method	Buffer	Sample amount
ds339	Poly-L-lysine (PLL)	50 mM TRIS, 150 mM NaCl, pH 7.4	7 ng ds339
ds339	Poly-L-ornithine (PLO)	50 mM TRIS, 150 mM NaCl, pH 7.4	10 ng ds339
ds339	Mg^{2+}/ Ni^{2+}	25 mM $MgCl_2$, 20 mM HEPES, 10 mM NaCl exchange for 3 mM $NiCl_2$, 20 mM HEPES, 10 mM NaCl, pH 7.4	7 ng ds339
ds339	Ni^{2+}	3 mM $NiCl_2$, 20 mM HEPES, 10 mM NaCl, pH 7.4	6 ng ds339

ds339 + NDP52-FL	PLL	50 mM TRIS, 150 mM NaCl, pH 7.4	10 ng ds339, 6 ng NDP52-FL
ds339 + NDP52-FL	PLO	50 mM TRIS, 150 mM NaCl, pH 7.4	10 ng ds339, 40 ng NDP52- FL
ds339 + NDP52-FL	Mg^{2+}/ Ni^{2+}	25 mM MgCl2, 20 mM HEPES, 10 mM NaCl exchange for 3 mM $NiCl_2$, 20 mM HEPES, 10 mM NaCl, pH 7.4	7 ng ds339, 4 ng NDP52-FL
ds339 + NDP52-FL	Ni^{2+}	3 mM $NiCl_2$, 20 mM HEPES, 10 mM NaCl, pH 7.4	7 ng ds339, 10 ng NDP52-FL
ds339 + cNDP52	PLL	50 mM TRIS, 150 mM NaCl, pH 7.4	14 ng ds339, 10 ng cNDP52
NDP52- FL	PLL	50 mM TRIS, 150 mM NaCl, pH 7.4	3- 8 ng NDP52- FL
NDP52- FL	PLO	50 mM TRIS, 150 mM NaCl, pH 7.4	10 ng NDP52- FL
NDP52- FL	Mg^{2+}/ Ni^{2+}	25 mM MgCl2, 20 mM HEPES, 10 mM NaCl exchange for 3 mM NiCl2, 20 mM HEPES, 10 mM NaCl, pH 7.4	24 ng NDP52- FL
NDP52- FL	Ni^{2+}	3 mM NiCl2, 50 mM TRIS, 150 mM NaCl or 3 mM $NiCl_2$, 20 mM HEPES, 10 mM NaCl, pH 7.4	7 ng or 16 ng NDP52- FL
cNDP52	PLL	50 mM TRIS, 150 mM NaCl, pH 7.4	3- 8 ng cNDP52

Supplementary Table 5 – HeLa RNA-Seq enriched GO Biological processes

p_{adj}	Gene count	Pathways
6.12E-13	157	Response to organic substance
1.23E-11	149	Regulation of multicellular organismal process
6.06E-11	126	Anatomical structure morphogenesis
6.06E-11	46	Positive regulation of cell migration
9.75E-11	61	Regulation of cell migration
9.75E-11	65	Regulation of locomotion
9.75E-11	47	Positive regulation of locomotion
9.75E-11	47	Positive regulation of cellular component movement
1.34E-10	104	Tissue development
2.83E-10	62	Regulation of cell motility
3.50E-10	123	Regulation of developmental process
3.74E-10	65	Regulation of cellular component movement
6.00E-10	151	Animal organ development
8.97E-10	94	Locomotion
0.000123	64	Circulatory system development
0.000173	5	Peptide cross-linking via chondroitin 4-sulfate glycosaminoglycan
0.000219	33	Extracellular matrix organization
0.000362	46	Vasculature development
0.000963	121	Anatomical structure morphogenesis
0.001308	117	Regulation of transcription by RNA polymerase II
0.00131	100	Movement of cell or subcellular component
0.001842	121	Transcription by RNA polymerase II
0.001842	58	Enzyme linked receptor protein signaling pathway
0.001842	44	Transmembrane receptor protein tyrosine kinase signaling pathway
0.002537	88	Locomotion
0.003258	41	Blood vessel development
0.003744	149	Regulation of transcription, DNA-templated

Supplementary Table 6 – MCF-7 RNA-Seq enriched GO Biological processes

p_{adj}	Gene count	Pathways
7.33E-05	27	Connective tissue development
0.000519	49	Transmembrane receptor protein tyrosine kinase signaling pathway
0.00239	6	Ganglioside catabolic process
0.00239	20	Cartilage development
0.002642	78	Lipid metabolic process
0.002642	103	Movement of cell or subcellular component
0.002642	20	Inositol lipid-mediated signaling
0.002904	52	Regulation of cell migration
0.002904	4	Nucleoside bisphosphate catabolic process
0.002904	98	Small molecule metabolic process
0.002904	58	Regulation of cellular component movement
0.002955	19	Phosphatidylinositol-mediated signaling
0.002955	83	Cell motility
2.69E-16	100	Cell cycle
4.74E-16	84	Cell cycle process
7.62E-14	61	Mitotic cell cycle process
7.97E-14	66	Mitotic cell cycle
2.62E-11	45	Cell division
7.52E-11	32	Chromosome segregation
4.30E-10	65	Regulation of cell cycle
7.62E-09	22	Sister chromatid segregation
9.57E-09	26	Nuclear chromosome segregation
2.36E-08	33	Nuclear division
5.40E-08	26	Mitotic nuclear division
7.25E-08	25	DNA replication
7.25E-08	34	Organelle fission
8.16E-08	19	Mitotic sister chromatid segregation
2.36E-07	37	Mitotic cell cycle phase transition

Supplementary Table 7 – Enriched GO Biological processes from recombinant NDP52-FL nuclear interactome

Fold Enrichment	Genes in list	FDR	Functional Category – Biological Process
4.75	11	0.0142	DNA geometric change (GO:0032392)
3.8	10	0.0315	DNA duplex unwinding (GO:0032508)
3.03	12	0.0407	establishment of cell polarity (GO:0030010)
2.86	24	0.00249	regulation of small GTPase mediated signal transduction (GO:0051056)
2.7	56	3.17E-08	microtubule-based process (GO:0007017)
2.62	36	2.63E-04	microtubule cytoskeleton organization (GO:0000226)
2.6	25	0.01	positive regulation of proteolysis (GO:0045862)
2.58	38	2.40E-04	cellular component morphogenesis (GO:0032989)
2.57	32	0.00194	cell part morphogenesis (GO:0032990)
2.54	30	0.00305	neuron projection morphogenesis (GO:0048812)
2.52	30	0.00511	plasma membrane bounded cell projection morphogenesis (GO:0120039)
2.52	30	0.00535	cell projection morphogenesis (GO:0048858)
2.48	23	0.0303	microtubule-based movement (GO:0007018)
2.47	26	0.0197	cell morphogenesis involved in neuron differentiation (GO:0048667)
2.43	74	1.22E-08	cytoskeleton organization (GO:0007010)
2.41	37	0.00142	actin filament-based process (GO:0030029)
2.34	24	0.0411	plasma membrane bounded cell projection assembly (GO:0120031)
2.26	32	0.0102	actin cytoskeleton organization (GO:0030036)
2.26	65	3.72E-06	cell projection organization (GO:0030030)
2.23	30	0.023	cell morphogenesis involved in differentiation (GO:0000904)
2.18	61	2.57E-05	plasma membrane bounded cell projection organization (GO:0120036)
2.15	29	0.0413	supramolecular fiber organization (GO:0097435)
2.14	36	0.0119	neuron projection development (GO:0031175)
2.13	35	0.0166	protein localization to organelle (GO:0033365)
2.13	37	0.0143	cell morphogenesis (GO:0000902)
2.12	44	0.00294	cell cycle process (GO:0022402)
2.07	32	0.0353	positive regulation of hydrolase activity (GO:0051345)
2.03	43	0.0068	neuron development (GO:0048666)
2.01	63	2.44E-04	cell cycle (GO:0007049)
2.01	38	0.028	organelle assembly (GO:0070925)
1.99	173	4.25E-16	organelle organization (GO:0006996)
1.96	52	0.00292	chromosome organization (GO:0051276)

1.93	52	0.00423	regulation of hydrolase activity (GO:0051336)
1.87	70	2.45E-04	movement of cell or subcellular component (GO:0006928)
1.87	66	0.00143	cellular protein localization (GO:0034613)
1.86	46	0.0318	biological adhesion (GO:0022610)
1.84	66	0.00211	cellular macromolecule localization (GO:0070727)
1.84	45	0.0404	cell adhesion (GO:0007155)
1.83	45	0.0397	regulation of cellular component biogenesis (GO:0044087)
1.83	56	0.00954	regulation of organelle organization (GO:0033043)
1.81	56	0.0094	protein-containing complex assembly (GO:0065003)
1.79	105	6.16E-06	cellular component assembly (GO:0022607)
1.79	243	1.15E-18	cellular component organization (GO:0016043)
1.77	114	2.75E-06	cellular component biogenesis (GO:0044085)
1.75	61	0.0108	protein-containing complex subunit organization (GO:0043933)
1.7	247	2.10E-18	cellular component organization or biogenesis (GO:0071840)
1.66	83	0.00228	protein localization (GO:0008104)
1.65	69	0.0208	cell development (GO:0048468)
1.6	96	0.00143	cellular localization (GO:0051641)
1.6	97	0.00299	regulation of cellular component organization (GO:0051128)
1.59	90	0.00614	nervous system development (GO:0007399)
1.51	98	0.00314	regulation of catalytic activity (GO:0050790)
1.5	119	0.00295	regulation of molecular function (GO:0065009)

Supplementary Table 8 – Enriched GO Molecular Functions from recombinant NDP52-FL nuclear interactome

Fold Enrichment	Genes in list	FDR	Functional Category – Molecular Function
29.2	3	0.0468	pantothenate kinase activity (GO:0004594)
7.08	6	0.0372	nucleosome-dependent ATPase activity (GO:0070615)
4.8	9	0.0213	DNA helicase activity (GO:0003678)
4.47	10	0.0173	beta-catenin binding (GO:0008013)
4.41	12	0.00517	ATPase, acting on DNA (GO:0008094)
3.74	12	0.0182	single-stranded DNA binding (GO:0003697)
3.7	15	0.00408	helicase activity (GO:0004386)
3.66	11	0.0373	cytoskeletal motor activity (GO:0003774)
3.2	19	2.85E-03	catalytic activity, acting on DNA (GO:0140097)
3.2	23	4.85E-04	ATP hydrolysis activity (GO:0016887)
3.13	16	0.0125	phosphatase binding (GO:0019902)
2.8	22	4.03E-03	mRNA binding (GO:0003729)
2.76	33	1.01E-04	GTPase activator activity (GO:0005096)
2.74	36	4.11E-05	ATP-dependent activity (GO:0140657)
2.68	34	1.14E-04	GTPase regulator activity (GO:0030695)
2.68	34	1.09E-04	nucleoside-triphosphatase regulator activity (GO:0060589)
2.59	18	0.0366	microtubule binding (GO:0008017)
2.53	21	0.0184	cadherin binding (GO:0045296)
2.39	33	0.00139	cell adhesion molecule binding (GO:0050839)
2.18	41	0.00144	enzyme activator activity (GO:0008047)
2.16	33	0.00824	catalytic activity, acting on a nucleic acid (GO:0140640)
2.15	25	0.0483	actin binding (GO:0003779)
2.14	39	2.85E-03	structural molecule activity (GO:0005198)
2.14	55	6.53E-05	cytoskeletal protein binding (GO:0008092)
2.07	83	9.42E-07	adenyl ribonucleotide binding (GO:0032559)
2.06	79	1.52E-06	ATP binding (GO:0005524)
2.05	83	9.16E-07	adenyl nucleotide binding (GO:0030554)
2	32	3.13E-02	nucleoside-triphosphatase activity (GO:0017111)
1.99	86	1.63E-06	RNA binding (GO:0003723)
1.85	91	9.13E-06	purine ribonucleotide binding (GO:0032555)
1.84	87	1.97E-05	purine ribonucleoside triphosphate binding (GO:0035639)

1.84	91	1.37E-05	purine nucleotide binding (GO:0017076)
1.84	91	1.30E-05	ribonucleotide binding (GO:0032553)
1.84	62	0.00117	protein-containing complex binding (GO:0044877)
1.81	58	2.94E-03	enzyme regulator activity (GO:0030234)
1.78	104	6.38E-06	carbohydrate derivative binding (GO:0097367)
1.76	98	2.05E-05	nucleotide binding (GO:0000166)
1.75	98	1.96E-05	nucleoside phosphate binding (GO:1901265)
1.64	87	1.29E-03	enzyme binding (GO:0019899)
1.62	100	4.22E-04	anion binding (GO:0043168)
1.61	104	2.46E-04	small molecule binding (GO:0036094)
1.49	153	5.15E-05	nucleic acid binding (GO:0003676)
1.46	223	3.07E-07	heterocyclic compound binding (GO:1901363)
1.45	225	2.42E-07	organic cyclic compound binding (GO:0097159)
1.27	198	1.24E-02	ion binding (GO:0043167)
1.17	432	7.60E-07	protein binding (GO:0005515)
0.43	18	0.0057	molecular transducer activity (GO:0060089)
0.43	18	5.56E-03	signaling receptor activity (GO:0038023)
0.36	13	2.75E-03	transmembrane signaling receptor activity (GO:0004888)
0.09	2	1.92E-05	G protein-coupled receptor activity (GO:0004930)

Supplementary Table 9 – Enriched GO Biological Processes from recombinant CoCoA nuclear interactome

Fold Enrichment	Genes in list	FDR	Functional Category – Biological Process
40.3	3	0.0489	pre-replicative complex assembly involved in cell cycle DNA replication (GO:1902299)
40.3	3	0.0478	pre-replicative complex assembly involved in nuclear cell cycle DNA replication (GO:0006267)
40.3	3	0.0467	pre-replicative complex assembly (GO:0036388)
17.1	4	0.0498	positive regulation of mRNA splicing, via spliceosome (GO:0048026)
9.17	8	0.00699	DNA duplex unwinding (GO:0032508)
8.55	8	0.0089	DNA geometric change (GO:0032392)
7.3	8	0.0191	regulation of mRNA splicing, via spliceosome (GO:0048024)
6.27	10	0.00944	regulation of RNA splicing (GO:0043484)
3.88	12	0.0398	regulation of mRNA metabolic process (GO:1903311)
3.15	16	0.034	DNA repair (GO:0006281)
2.84	17	0.0491	mRNA metabolic process (GO:0016071)
2.63	20	0.0415	DNA metabolic process (GO:0006259)
2.55	23	0.0295	RNA processing (GO:0006396)
2.37	24	0.049	regulation of cellular component biogenesis (GO:0044087)
2.25	48	0.00101	nucleic acid metabolic process (GO:0090304)
2.21	33	0.0182	RNA metabolic process (GO:0016070)
2.17	36	0.0159	negative regulation of macromolecule biosynthetic process (GO:0010558)
2.13	35	0.0205	negative regulation of cellular macromolecule biosynthetic process (GO:2000113)
2.11	37	0.0164	negative regulation of biosynthetic process (GO:0009890)
2.09	36	0.0201	negative regulation of cellular biosynthetic process (GO:0031327)
2.09	55	0.00169	nucleobase-containing compound metabolic process (GO:0006139)
1.99	57	0.0012	cellular aromatic compound metabolic process (GO:0006725)
1.98	42	0.0165	gene expression (GO:0010467)
1.97	55	0.00232	heterocycle metabolic process (GO:0046483)
1.96	61	8.69E-04	organic cyclic compound metabolic process (GO:1901360)
1.86	63	0.00204	cellular nitrogen compound metabolic process (GO:0034641)
1.83	46	0.0341	regulation of cellular component organization (GO:0051128)
1.82	65	0.00189	organelle organization (GO:0006996)
1.82	57	0.00862	negative regulation of metabolic process (GO:0009892)
1.75	51	0.0312	negative regulation of macromolecule metabolic process (GO:0010605)
1.65	65	0.0199	regulation of RNA metabolic process (GO:0051252)

1.63	68	0.02	regulation of nucleobase-containing compound metabolic process (GO:0019219)
1.61	67	0.0273	regulation of cellular macromolecule biosynthetic process (GO:2000112)
1.6	67	0.0331	regulation of macromolecule biosynthetic process (GO:0010556)
1.59	69	0.0327	regulation of cellular biosynthetic process (GO:0031326)
1.58	92	0.00213	cellular component organization or biogenesis (GO:0071840)
1.56	88	0.00597	cellular component organization (GO:0016043)
1.5	76	0.0424	regulation of gene expression (GO:0010468)

Supplementary Table 10 – Enriched GO Molecular Functions from recombinant CoCoA nuclear interactome

Fold Enrichment	Genes in list	FDR	Functional Category – Biological Process
9.02	7	0.0035	DNA helicase activity (GO:0003678)
8.87	10	1.14E-04	ATPase, acting on DNA (GO:0008094)
6.55	11	4.08E-04	helicase activity (GO:0004386)
5.71	17	1.49E-05	ATP hydrolysis activity (GO:0016887)
5.29	13	4.30E-04	catalytic activity, acting on DNA (GO:0140097)
4.59	25	5.28E-07	ATP-dependent activity (GO:0140657)
4.3	14	0.00157	mRNA binding (GO:0003729)
3.48	22	1.61E-04	catalytic activity, acting on a nucleic acid (GO:0140640)
3.18	21	9.63E-04	nucleoside-triphosphatase activity (GO:0017111)
2.9	21	0.00322	pyrophosphatase activity (GO:0016462)
2.88	21	0.00338	hydrolase activity, acting on acid anhydrides, in phosphorus-containing anhydrides (GO:0016818)
2.88	21	0.00326	hydrolase activity, acting on acid anhydrides (GO:0016817)
2.79	50	6.17E-08	RNA binding (GO:0003723)
2.58	41	1.93E-05	ATP binding (GO:0005524)
2.47	41	4.09E-05	adenyl ribonucleotide binding (GO:0032559)
2.45	41	4.43E-05	adenyl nucleotide binding (GO:0030554)
2.35	46	3.13E-05	purine ribonucleoside triphosphate binding (GO:0035639)
2.29	47	3.42E-05	ribonucleotide binding (GO:0032553)
2.26	46	7.87E-05	purine ribonucleotide binding (GO:0032555)
2.25	52	1.73E-05	nucleotide binding (GO:0000166)
2.25	52	1.55E-05	nucleoside phosphate binding (GO:1901265)
2.24	46	8.08E-05	purine nucleotide binding (GO:0017076)
2.15	55	2.07E-05	anion binding (GO:0043168)
2.06	55	7.10E-05	small molecule binding (GO:0036094)
2	85	5.46E-08	nucleic acid binding (GO:0003676)
1.94	47	0.00209	carbohydrate derivative binding (GO:0097367)
1.79	113	1.12E-08	heterocyclic compound binding (GO:1901363)
1.76	113	1.86E-08	organic cyclic compound binding (GO:0097159)
1.21	185	1.48E-04	protein binding (GO:0005515)

Supplementary Table 11 – Enriched GO Biological processes from endogenous NDP52 interactome in non-treated HeLa cells

Fold Enrichment	Genes in list	FDR	Functional Category – Biological Process
2.77	24	0.00874	ncRNA metabolic process (GO:0034660)
2.68	21	0.0354	ribonucleoprotein complex biogenesis (GO:0022613)
2.61	25	0.0127	supramolecular fiber organization (GO:0097435)
2.3	36	0.00466	RNA processing (GO:0006396)
2	52	0.00234	RNA metabolic process (GO:0016070)
1.97	73	7.16E-5	nucleic acid metabolic process (GO:0090304)
1.95	45	0.0179	intracellular transport (GO:0046907)
1.93	49	0.0117	cellular protein localization (GO:0034613)
1.91	49	0.0122	cellular macromolecule localization (GO:0070727)
1.91	45	0.0223	establishment of protein localization (GO:0045184)
1.87	69	5.52E-4	gene expression (GO:0010467)
1.84	84	8.18E-5	nucleobase-containing compound metabolic process (GO:0006139)
1.82	64	0.00324	protein localization (GO:0008104)
1.81	88	9.04E-5	heterocycle metabolic process (GO:0046483)
1.8	77	5.44E-4	macromolecule localization (GO:0033036)
1.79	75	9.15E-4	cellular localization (GO:0051641)
1.77	88	1.38E-4	cellular aromatic compound metabolic process (GO:0006725)
1.71	101	8.49E-5	cellular nitrogen compound metabolic process (GO:0034641)
1.7	92	3.51E-4	organic cyclic compound metabolic process (GO:1901360)
1.62	71	0.0248	regulation of cellular component organization (GO:0051128)
1.59	73	0.0459	cellular component biogenesis (GO:0044085)
1.56	158	1.03E-5	cellular component organization or biogenesis (GO:0071840)
1.54	151	3.12E-5	cellular component organization (GO:0016043)
1.51	160	2.61E-5	macromolecule metabolic process (GO:0043170)

Supplementary Table 12 – Enriched GO Biological processes from endogenous NDP52 interactome in HeLa cells following α-amanitin treatment

Fold Enrichment	Genes in list	FDR	Functional Category – Biological Process
7.49	7	0.0339	mitochondrion localization (GO:0051646)
5	10	0.0279	regulation of DNA replication (GO:0006275)
4.39	11	0.0339	signal transduction in response to DNA damage (GO:0042770)
3.65	25	1.66E-4	regulation of cell cycle phase transition (GO:1901987)
3.28	17	0.0205	regulation of mitotic cell cycle phase transition (GO:1901990)
2.86	32	4.76E-4	mitotic cell cycle (GO:0000278)
2.73	26	0.00629	mitotic cell cycle process (GO:1903047)
2.73	32	8.54E-4	regulation of cell cycle process (GO:0010564)
2.72	22	0.0214	regulation of mitotic cell cycle (GO:0007346)
2.7	24	0.0142	cell junction organization (GO:0034330)
2.53	34	0.00241	cellular response to DNA damage stimulus (GO:0006974)
2.52	30	0.00806	protein localization to organelle (GO:0033365)
2.35	44	5.72E-4	regulation of cell cycle (GO:0051726)
2.31	44	7.19E-4	chromosome organization (GO:0051276)
2.19	33	0.0276	cell cycle process (GO:0022402)
2.17	49	8.66E-4	cell cycle (GO:0007049)
1.99	50	0.00578	protein-containing complex subunit organization (GO:0043933)
1.93	43	0.029	protein-containing complex assembly (GO:0065003)
1.89	49	0.0162	cellular macromolecule localization (GO:0070727)
1.88	42	0.0468	negative regulation of signal transduction (GO:0009968)
1.87	48	0.0282	cellular protein localization (GO:0034613)
1.82	114	5.61E-7	organelle organization (GO:0006996)
1.79	49	0.0389	intracellular signal transduction (GO:0035556)
1.79	67	0.00423	nucleic acid metabolic process (GO:0090304)
1.76	50	0.0479	cellular response to stress (GO:0033554)
1.74	179	2.7E-12	cellular component organization or biogenesis (GO:0071840)
1.73	73	0.00542	cellular localization (GO:0051641)
1.72	80	0.00233	cellular component biogenesis (GO:0044085)
1.72	170	7.99E-11	cellular component organization (GO:0016043)
1.68	71	0.0129	cellular component assembly (GO:0022607)
1.6	74	0.028	nucleobase-containing compound metabolic process (GO:0006139)
