# Supplementary material for: Autophagy receptor NDP52 alters DNA conformation to modulate RNA polymerase II transcription
Source: Nat Commun. 2023 May 18;14:2855. doi: 10.1038/s41467-023-38572-9 (PMC10195817; doi:10.1038/s41467-023-38572-9)
Supplement: Supplementary file 2 — Reporting Summary [file 41467_2023_38572_MOESM2_ESM.pdf]

Corresponding author(s): Christopher P. Toseland
Last updated by author(s): 29-03-2023

Reporting Summary

Nature Research wishes to improve the reproducibility of the work that we publish. This form provides structure for consistency and transparency in reporting. For further information on Nature Research policies, see our Editorial Policies and the Editorial Policy Checklist.

Statistics

For all statistical analyses, confirm that the following items are present in the figure legend, table legend, main text, or Methods section.

n/a	Confirmed	
☐	☒	The exact sample size (*n*) for each experimental group/condition, given as a discrete number and unit of measurement
☐	☒	A statement on whether measurements were taken from distinct samples or whether the same sample was measured repeatedly
☐	☒	The statistical test(s) used AND whether they are one- or two-sided<br>*Only common tests should be described solely by name; describe more complex techniques in the Methods section.*
☒	☐	A description of all covariates tested
☒	☐	A description of any assumptions or corrections, such as tests of normality and adjustment for multiple comparisons
☐	☒	A full description of the statistical parameters including central tendency (e.g. means) or other basic estimates (e.g. regression coefficient) AND variation (e.g. standard deviation) or associated estimates of uncertainty (e.g. confidence intervals)
☐	☒	For null hypothesis testing, the test statistic (e.g. *F*, *t*, *r*) with confidence intervals, effect sizes, degrees of freedom and *P* value noted<br>*Give P values as exact values whenever suitable.*
☒	☐	For Bayesian analysis, information on the choice of priors and Markov chain Monte Carlo settings
☒	☐	For hierarchical and complex designs, identification of the appropriate level for tests and full reporting of outcomes
☒	☐	Estimates of effect sizes (e.g. Cohen's *d*, Pearson's *r*), indicating how they were calculated

Our web collection on statistics for biologists contains articles on many of the points above.

Software and code

Policy information about availability of computer code

Data collection	Commercial microscopy software (Zeiss ZEN Black v2.3) was used to acquire STORM and confocal data. Nikon instruments (NIS-Elements v3.7) software was used to gather single molecule data. AFM data was collected using Bruker Nanoscope software (v5.1). Biochemical Data was collected using commercial BMG-Labtech software running Clariostar plate reader system. SECMALS data were gathered using OMNISEC v8. DLS was gathered using Zetasizer software (v3).
Data analysis	Zeiss ZEN Black (v2.3) was used to process the STORM data. All images were exported as TIFFs. STORM localisation tables were exported as text files for input in to open software (Clus-DoC). Single molecule tracking data was acquired in nd2 format and then exported as TIFFs. Tracking files are saved as text files. EasyFRAP was used to measure FRAP parameters. AFM image analysis was performed using TopoStats. ImageJ (V1.53K) was used to produce final images. Proteomics data was analysed using Progenesis QI (Non Linear dynamics) and outputs were saved as text files. RNA-seq data was processed using Trimmomatic v.0.36, STAR aligner v.2.5.2b, Subread package v.1.5.2 and iDEP93. SECMALS was analysed using OMNISEC v8. DLS was analyzed using Zetasizer software (v3).

For manuscripts utilizing custom algorithms or software that are central to the research but not yet described in published literature, software must be made available to editors and reviewers. We strongly encourage code deposition in a community repository (e.g. GitHub). See the Nature Research guidelines for submitting code & software for further information.

Data

Policy information about availability of data

All manuscripts must include a data availability statement. This statement should provide the following information, where applicable:

- Accession codes, unique identifiers, or web links for publicly available datasets
- A list of figures that have associated raw data
- A description of any restrictions on data availability

Data are contained within the Source File. All raw image data are available upon request from the corresponding author. The mass spectrometry proteomics data have been deposited to the ProteomeXchange Consortium with the data identifier PXD030238 (http://proteomecentral.proteomexchange.org/cgi/GetDataset?ID=PXD030238). RNA-Seq data were deposited in the Gene Expression Omnibus (GEO) database under the accession number GSE188567 (https://www.ncbi.nlm.nih.gov/geo/query/acc.cgi?acc=GSE188567).

The Homo sapiens GRCh38 reference genome (https://www.ncbi.nlm.nih.gov/assembly/GCF_000001405.26/) was used for analysing the RNA-seq data.

Field-specific reporting

Please select the one below that is the best fit for your research. If you are not sure, read the appropriate sections before making your selection.

☒ Life sciences ☐ Behavioural & social sciences ☐ Ecological, evolutionary & environmental sciences

For a reference copy of the document with all sections, see nature.com/documents/nr-reporting-summary-flat.pdf

Life sciences study design

All studies must disclose on these points even when the disclosure is negative.

Sample size	Besides microscopy, all experiments were performed in triplicate or greater. STORM images were performed on a minimum of 10 cells per condition. Single molecule tracking experiments were performed on a minimum of 100 cells per condition. The microscopy numbers were determined prior to experiment based on timing/scale of the specific experiment. Post-determination was then applied by measuring statistic differences between the treatments where more experiments would be performed if variance prevented data determination. The variance did not impact measurement conclusion therefore the sample sizes were used.<br><br>Sample sizes for other experiments were not Pre-calculated. The experiments were performed as three-independent measurements.
Data exclusions	None
Replication	Imaging experiments were repeated on multiple occasions (at least 4 times). RNA-seq experiments were performed in triplicate but not repeated independently due to costs. All other experiments were repeated in triplicate. All replicates were successful. Proteomics experiments were performed as three biological replicates.
Randomization	Conditions were grouped based on treatments and experiment group.
Blinding	Imaging experiments were not blinded because phenotypic changes were clear (e.g. lack of protein due to knockdown). Other experiments were blinded.

Reporting for specific materials, systems and methods

We require information from authors about some types of materials, experimental systems and methods used in many studies. Here, indicate whether each material, system or method listed is relevant to your study. If you are not sure if a list item applies to your research, read the appropriate section before selecting a response.

Materials & experimental systems

n/a	Involved in the study	
☐	☒	Antibodies
☐	☒	Eukaryotic cell lines
☒	☐	Palaeontology and archaeology
☒	☐	Animals and other organisms
☒	☐	Human research participants
☒	☐	Clinical data
☒	☐	Dual use research of concern

Methods

n/a	Involved in the study	
☒	☐	ChIP-seq
☒	☐	Flow cytometry
☒	☐	MRI-based neuroimaging

Antibodies

Antibodies used	Rabbit anti-NDP52 (Genetex GTX115378), Mouse anti-RNAPII phospho Ser5 (Abcam Ab5408), Donkey anti-rabbit Alexa Fluor 647-conjugated (Abcam Ab181347), Donkey anti-mouse Alexa Fluor 488-conjugated (Abcam Ab181289), Mouse anti-actin (Abcam, ab6276) and Goat anti-rabbit IgG H&L coupled to horseradish peroxidase (Abcam, ab6721).
Validation	Target Validation has been performed by the suppliers: ab5408 by alkaline phosphatase treatment and peptide binding assays. ab6276 through knockout in mammalian cell lines. GTX115378 has been validated within this work using siRNA knockdown. Secondary antibodies were validated for non-specific interactions by performing experiments in the absence of primary antibody.

Eukaryotic cell lines

Policy information about cell lines

Cell line source(s)	HeLa (ECACC 93021013) and MCF-7 (ECACC 86012803)
Authentication	Authenticated by supplier through STR-PCR and Karyotype.
Mycoplasma contamination	Not detected upon testing.
Commonly misidentified lines (See ICLAC register)	No commonly misidentified cell lines were used in this study.
